# Identification of Key Genes Associated with Endothelial Cell Dysfunction in Atherosclerosis Using Multiple Bioinformatics Tools

**DOI:** 10.1155/2022/5544276

**Published:** 2022-01-10

**Authors:** Guofu Zhang, Hui Yu, Jingjing Su, Chao Chi, Lide Su, Fenglin Wang, Ying Zheng, Baodong Xie, Kai Kang

**Affiliations:** ^1^Department of Cardiovascular Surgery, The First Affiliated Hospital of Harbin Medical University, Harbin, Heilongjiang Province, China; ^2^Future Laboratory of the Second Affiliated Hospital of Harbin Medical University, Harbin, Heilongjiang Province, China; ^3^NHC Key Laboratory of Cell Transplantation, The First Affiliated Hospital Harbin Medical University, 23 Youzheng Street, Nangang District, Harbin, Heilongjiang Province, China

## Abstract

Atherosclerosis is the most notable cardiovascular disease, the latter being the main cause of death globally. Endothelial cell dysfunction plays a major role in the pathogenesis of atherosclerosis. However, it is currently unclear which genes are involved between endothelial cell dysfunction and atherosclerosis. This study was aimed at identifying these genes. Based on the GSE83500 dataset, the quantification of endothelial cell function was conducted using single-sample gene set enrichment analysis; the coexpression modules were conducted using weighted correlation network analysis. After building module-trait relationships, tan and yellow modules were regarded as hub modules. 10 hub genes from each hub module were identified by the protein-protein interaction network analysis. The key genes (*RAB5A*, *CTTN*, *ITGB1*, and *MMP9*) were obtained by comparing the expression differences of the hub gene between atherosclerotic and normal groups from the GSE28829 and GSE43292 datasets, respectively. ROC analysis showed the diagnostic value of key genes. Moreover, the differential expression of key genes in normal and atherosclerotic aortic walls was verified. *In vitro*, we establish a model of ox-LDL-injured endothelial cells and transfect *RAB5A* overexpression and shRNA plasmids. The results showed that overexpression of *RAB5A* ameliorates the proliferation and migration function of ox-LDL-injured endothelial cells, including the ability of tubule formation. It was speculated that the interferon response, Notch signaling pathways, etc. were involved in this function of *RAB5A* by using gene set variation analysis. With the multiple bioinformatics analysis methods, we detected that yellow and tan modules are related to the abnormal proliferation and migration of endothelial cells associated with atherosclerosis. *RAB5A*, *CTTN*, *ITGB1*, and *MMP9* can be used as potential targets for therapy and diagnostic markers. *In vitro*, overexpression of *RAB5A* can ameliorate the proliferation and migration function of ox-LDL-injured endothelial cells, and the possible molecules involved in this process were speculated.

## 1. Introduction

Having multiple pathogenic factors, cardiovascular disease is an important cause of death worldwide [1, 2]. Atherosclerosis is the main pathological change in cardiovascular disease. Because the endothelium is the interface between blood flow and the vessel wall, changes in endothelial cells are believed to play a major role in the pathogenesis of atherosclerosis [3]. Endothelial cell dysfunction (ECD) encompasses all maladaptive changes in the functional phenotype of endothelial cells and can lead to the earliest detectable changes in the course of atherosclerosis, that is, the local infiltration, capture, and chemical modification of circulating lipoprotein particles in the subendothelial space [4, 5]. It is widely accepted that there are several genes involved in the molecular mechanism of atherosclerosis and ECD. ECD usually occurs before atherosclerosis and induces abnormal proliferation and migration of endothelial cells identified in the early stage of atherosclerosis [6, 7]. Many experiments focused on understanding the role of ECD in the pathogenesis of atherosclerosis have obtained some meaningful results. To mention a few examples, Zhang et al. [8] reported that Hsa-Let-7g miRNA inhibits the apoptosis induced by oxidized low-density lipoprotein (ox-LDL) in endothelial cells by regulating the expression of CASP3. Similarly, Zhang et al. [9] found the essential roles of the E2F1/SNHG7/miR-186-5p/MMP2 axis in the proliferation and migration of endothelial cells, which provides a potential therapeutic target for atherosclerosis. However, the association between ECD and genes involved in atherosclerosis remains unclear. Because of the lack of clinical characteristic data of ECD, it is difficult to associate and analyze the changed gene expression with the changed cell function in the past. The emergence of single-sample gene set enrichment analysis (ssGSEA), weighted correlation network analysis (WGCNA), and Molecular Signatures Database makes this attempt possible.

In this study, ssGSEA, WGCNA, and other analysis methods were used to help us explore key genes that may be related to ECD associated with atherosclerosis. The ssGSEA is an extension of gene set enrichment analysis. It calculates the individual enrichment fractions of each pair of samples and gene sets. Each ssGSEA enrichment fraction represents the coordinated up- or downregulation of genes in a specific gene set in a sample. This algorithm has been successfully applied to the approximate calculation of immune cell infiltration levels in breast and gallbladder cancer, to name a few [10]. WGCNA is a common method for performing correlation network analysis, identifying disease-related gene modules, and determining the phenotypic traits of key genes [11]. It has been widely used in many biological analysis processes, including cancer and mouse genetics studies [12–14]. Moreover, it has been used with increased frequency to obtain gene correlation patterns of microarray samples. The WGCNA method considers transcriptome interactions by constructing coexpression modules, thereby contributing to the study of phenotypic traits and providing more in-depth analyses. Therefore, the important signaling pathways and genes related to corresponding phenotypic traits can be explored.

There are a large number of completed high-throughput sequencing of various diseases in the Gene Expression Omnibus (GEO) database. Therefore, we can use the GEO database, Molecular Signatures Database, and multiple bioinformatics analysis methods to explore the relationship between ECD and atherosclerosis-associated genes, which can provide us with potential targets to intervene in atherosclerosis. When we found key genes, further studies are nevertheless needed to elucidate the detailed biological function of these genes in the pathogenesis of atherosclerosis.

## 2. Material and Methods

### 2.1. Datasets

The GSE83500 dataset in the Gene Expression Omnibus (GEO) database was selected based on the GPL13667 platform to analyze the vessel walls of patients with atherosclerosis. The GPL platform includes data derived from frozen aortic tissue samples obtained from 17 MI patients and 20 non-MI patients. Then, the GSE28829 and GSE43292 datasets which involve mRNA expression data of normal and atherosclerotic plaque samples were downloaded to verify the expression of key genes and build a prediction model of atherosclerosis (the details of GSE83500, GSE28829, and GSE43292 datasets can be seen in Supplemental File 10).

### 2.2. Quantification of Endothelial Cell Function

The endothelial cell migration/proliferation dataset was predefined by the Molecular Signatures Database (v7.0), with their migration/proliferation levels in each sample quantified using the ssGSEA algorithm. The latter was determined through the sorting of markers.

### 2.3. Construction of Coexpression Gene Modules

The “WGCNA” R package was used to construct the coexpression network for all genes across 37 samples. Expression values of 10,000 genes were used to construct a coexpression network. Samples were used to calculate Pearson's correlation matrices. The weighted adjacency matrix was created with the formula amn = ∣cmn | *β*, where amn represents the adjacency between gene *m* and gene *n*, cmn represents Pearson's correlation of genes *m* and *n*, and *β* indicates the soft-power threshold. Furthermore, the weighted adjacency matrix was transformed into a topological overlap measure (TOM) matrix to estimate its connectivity property when the power of *β* is 5. Average linkage hierarchical clustering was used to construct a clustering dendrogram of the TOM matrix.

### 2.4. Construction of Module-Trait Relationships

Module eigengenes were used to perform component analysis of each module. The correlation between module eigengenes and the migration/proliferation function of endothelial cells was calculated to determine the significance of modules using Pearson's test. An individual module was considered significantly correlated with endothelial cell function when *p* < 0.01. The module (hub module) showing both significance and having the highest correlation coefficient was selected to calculate its module membership and gene significance for a trait. Module connectivity was defined as the absolute Pearson's correlation value between genes. Trait relationship was defined as the absolute Pearson's correlation value between each gene and the trait.

### 2.5. Identification of Hub Genes and Key Genes

The PPI network of the hub module genes was constructed using the STRING database and visualized through Cytoscape. The top 10 candidate genes were selected as the hub genes according to the degree of connectivity of each gene. To verify the accuracy of the identified hub genes, the expression of hub genes was detected in GSE28829 and GSE43292 datasets, respectively. Genes with significantly different expression profiles were considered key genes. ROC analysis was conducted to examine the performance of using key genes to predict atherosclerosis.

### 2.6. Functional Enrichment Analysis of Genes in the Hub Modules

To obtain the biological functions and signaling pathways involved in hub modules, the Metascape database was used for annotation and visualization. Gene Ontology (GO) and Kyoto Encyclopedia of Genes and Genomes (KEGG) pathway analyses were conducted for genes in hub modules. A minimum overlap ≥3 and *p* ≤ 0.01 were considered to be statistically significant.

### 2.7. Disease Correlation Analysis and Gene Set Variation Analysis

The first 20 atherosclerosis markers were identified from the GeneCards database (v5.0) and the correlation between the key genes and these markers analyzed. Gene set variation analysis (GSVA) is a nonparametric and unsupervised method for assessing transcriptome gene set enrichment. GSVA changes the gene level into the pathway level by comprehensively scoring the gene set of interest and then judging the biological function of the sample. In this study, gene sets were downloaded from the Molecular Signatures Database (v7.0) and the GSVA algorithm used to comprehensively score each gene set to evaluate the potential biological function changes across different samples.

### 2.8. Verify the Expression of Key Genes in Clinical Specimens

The samples of the atherosclerosis group were taken from the perforated tissue of the ascending aorta wall of patients who underwent coronary artery bypass grafting (CABG) in the Department of Cardiovascular Surgery of the First Affiliated Hospital of Harbin Medical University from March 2019 to June 2020; the samples of the normal group were taken from the discarded ascending aortic wall from patients who were undergoing aortic valve surgery at the same time, and patients with atherosclerosis were excluded. The study protocol has been approved by the Ethics Committee of the First Affiliated Hospital of Harbin Medical University, and informed consent was obtained from each patient. All aortic walls were soaked in RNAlater solution, and the intimal tissue was separated. It was immediately placed into liquid nitrogen for cooling and then transferred to a -80°C ultra-low-temperature refrigerator for storage. The total RNA was extracted, and Q-PCR was performed according to the protocol (Supplemental File 8). The primer sequences of key genes are shown in Table 1.

### 2.9. Establish a Model of ox-LDL-Injured HCEACs

Human coronary artery endothelial cells (HCAECs) (ScienCell, USA) were cultured in endothelial cell medium (ECM) containing 10% fetal bovine serum (FBS) and 5% endothelial cell growth factor (ECG) (ScienCell, USA). When the cells became 70–80% confluent, oxidized low-density lipoprotein (ox-LDL) (100 mg/L) was added to the medium. Cells were cultured for 24 hours to establish the model of ox-LDL-injured HCAECs. The morphological changes of cells were observed under an inverted microscope. The LDH activity of two groups was detected by using an LDH kit (Nanjing Jiancheng Bioengineering Institute, Jiangsu, China), which is to show whether the model is successful.

### 2.10. Preparation of *RAB5A* Overexpression and shRNA Plasmids and *In Vitro* Transfection

In this study, *RAB5A* overexpression and shRNA plasmids were designed by GenePharma (GeneChem, Shanghai, China) according to the manufacturer's instructions. HCAECs treated with ox-LDL were cultured in ECM containing 10% FBS and 5% ECG. The transfection process was carried out according to the instructions of Lipofectamine 2000 Reagent (Invitrogen, USA) (Supplemental File 9). HCAECs were cultured in 10% FBS-containing ECM until further analysis 48 h after transfection. HCAECs expressing green fluorescent protein (GFP) were then observed under a fluorescence microscope, and their transfection efficiency was determined by the ratio of GFP-positive cells to the total cell number. Finally, the expression of RAB5A in each group was measured by western blot. The protein content was quantified with a bicinchoninic acid protein (BCA) assay kit (Beyotime, Shanghai, China). After 10% SDS gel electrophoresis and membrane transfer, the membranes were blocked using 5% (*w*/*v*) nonfat milk diluted with Tris-buffered saline and Tween-20 (TBST). Then, the membranes were incubated with RAB5A Rabbit Anti-Human primary antibody (Affinity Biosciences, USA) at room temperature for 2 hours and HRP-Conjugated Goat Anti-Rabbit IgG(H+L) secondary antibody (Affinity Biosciences, USA) for 1 hour. Beyo ECL Plus (Beyotime, Shanghai, China) was used to detect blot results.

### 2.11. Endothelial Cell Proliferation and Cell Cycle Analysis

After ox-LDL treatment and plasmid transfection, cell proliferation of four groups (HCAECs, HCAECs-RAB5A overexpression, HCAECs-RAB5A shRNA, and HCAECs-NC) was detected by using CKK8 (tsbiochem, Shanghai, China) according to the manufacturer's procedures. Meanwhile, cell cycle analysis was performed on four group cells by using the Cell Cycle Detection Kit (Wanleibio, Shenyang, China). In brief, (1) collect cell samples. Digest and collect the digested cells into a centrifuge tube at 1000g for 5 min, and precipitate the cells. (2) Using phosphate buffer saline (PBS), resuspend the cells and adjust the cell concentration to 1 × 10^6^/mL at 1000g for 5 min, and precipitate cells. (3) Add 1 mL of 70% cold ethanol and fix it for 2 h to overnight. Wash the fixing solution with precooled PBS before dyeing. (4) Add 100 *μ*L RNase A to a 37°C water bath for 30 min. (5) Add 500 *μ*L propidium iodide (PI) staining solution to each tube and then mix evenly and keep it away from light at 4°C for 30 min. (6) Detect and analyze with a flow cytometer.

### 2.12. Endothelial Cell Apoptosis Detection

In order to analyze the apoptosis rate of cells from four groups, we performed Annexin-FITC/PI apoptosis detection according to the manufacturer's procedures (Annexin V-FITC/PI apoptosis detection kit, Solarbio, Beijing, China). The western blot test was used to detect apoptosis-related proteins, such as caspase-3, Bax, and Bcl2 (Wanleibio, Shenyang, China). *β*-Actin was used as a loading control. The process of western blot complies with the process mentioned above.

### 2.13. Wound Healing Assay

A total of 5 × 10^5^ cells from four groups were cultured in a six-well plate (Corning, New York, USA) with ECM containing 10% FBS for 24 hours. When the HCAECs became 80-90% confluent, cell monolayers were scratched in each well. Floating cells were washed away with PBS, and cells were incubated for 16 h with ECM serum-free medium in normal condition. Microscopic images (40x) were taken at 0 and 16 h.

### 2.14. Transwell Migration Assay

A Transwell upper chamber treated with 1% gelatin was balanced in the incubator for 1 h; then, 100 *μ*L serum-free ECM medium containing 5 × 10^4^ HCAECs from four groups was added. 600 *μ*L ECM contains 20% FBS which was added to the lower chamber to stimulate migration. Medium was discarded after cells were cultured for 24 hours. HCAECs were fixed with 10% formaldehyde solution; then, gently wipe off the upper nonmigrated cells. HCAECs were permeabilized for 15 minutes in 0.5% Triton-X-100 solution. Finally, DAPI staining solution was added. PBS rinsing is required before and after each step. HCAECs were observed under a fluorescence inverted microscope and photographed in 6 different locations.

### 2.15. Matrigel Tube Formation Assay

The 96-well plate was coated with 60 *μ*L/well of chilled Matrigel solution (10 mg/mL) without air bubbles and incubated for 1 h at 37°C to solidify. A total of 100 *μ*L ECM medium and 2‐3 × 10^4^ HCAECs from four groups were seeded in the 96-well plate. The endothelial tubule-like vascular network formation was observed after 4, 6, and 8 h of incubation and photographed in 6 different locations.

## 3. Results

### 3.1. Microarray Data Collection and Gene Expression Analysis

A 1total of 37 samples (17 MI and 20 non-MI) were included in the GSE83500 dataset. The GSE28829 dataset contains 29 atherosclerotic plaque samples (13 early and 16 advanced atherosclerotic plaque samples). The GSE43292 dataset contains 32 normal and 32 atherosclerotic plaque samples. The raw data was used to transform microarray information into gene expression data. Notably, probes matching several genes were eliminated, as were genes with a negative expression value. The average value was used as the expression data for genes matching more than a probe.

### 3.2. Construction and Selection of Coexpression Modules

**Figure 1:**
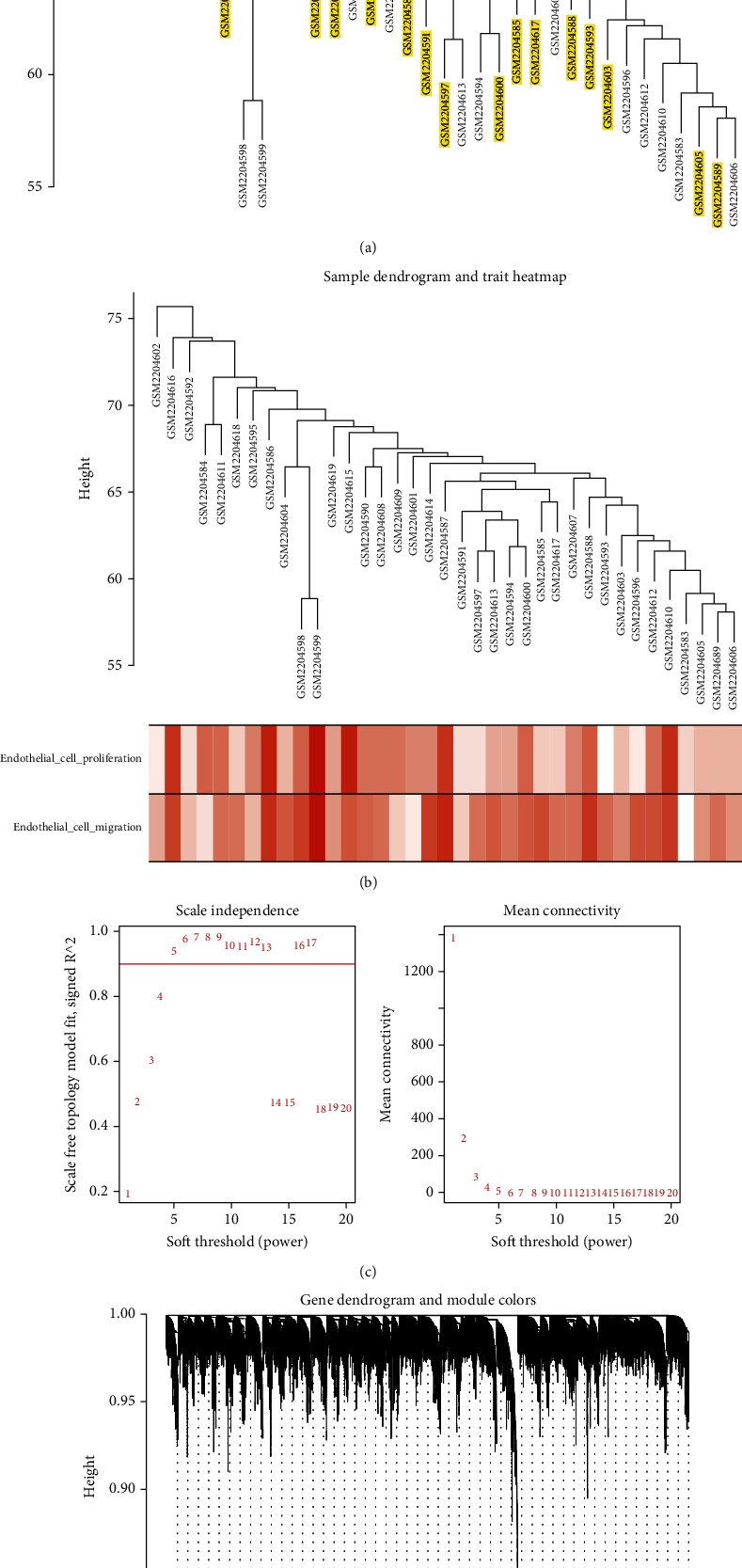
Construction of coexpression modules. (a) Sample clustering was conducted to detect outliers. Clusters, all passing the cutoff thresholds, were constructed from all samples. (b) Quantification of endothelial cell function by ssGSEA. The result is displayed as a trait heat map. (c) Analysis of network topology for various soft-thresholding powers. The left panel shows the scale-free fit index (*y*-axis) as a function of the soft-thresholding power (*x*-axis). The right panel displays the mean connectivity (*y*-axis) as a function of the soft-thresholding power (*x*-axis). (d) Gene clustering dendrograms. Dissimilarity was based on topological overlap together with assigned module colors. As a result, 32 coexpression modules were constructed and are shown in different colors. These modules were arranged from large to small based on the number of genes they included.

**Figure 2:**
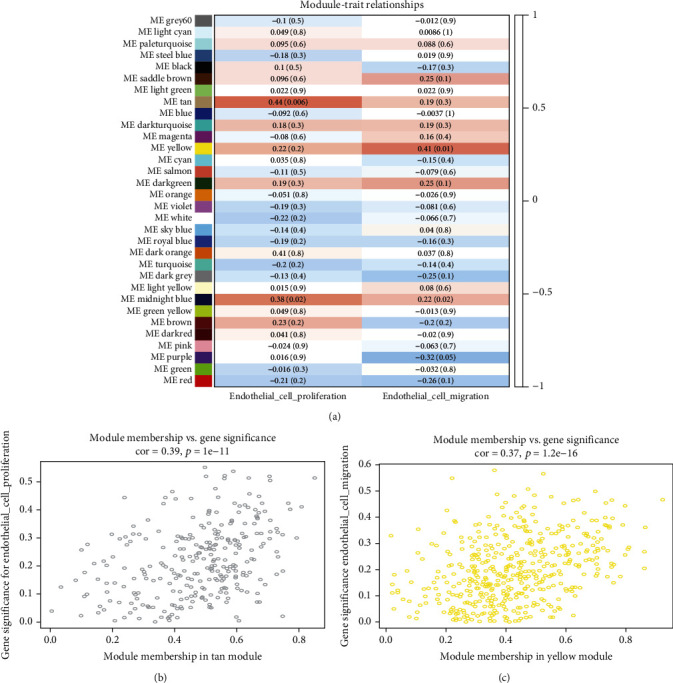
Module-trait associations and a scatter plot of the genes in the tan and yellow modules. (a) Module-trait associations. Each row corresponds to a module eigengene, while columns represent a trait. Each cell contains the corresponding correlation and *p* values. The table is color-coded according to the color legend. (b) Gene significance scatterplot for endothelial cell proliferation vs. module membership in the tan module (cor = 0.39, *p* = 1*e* − 11). (c) Gene significance scatterplots for endothelial cell migration vs. module membership in the yellow module (cor = 0.37, *p* = 1.2*e* − 16).

Following clustering analysis of samples, the ssGSEA program was used to quantify endothelial cell function and generate the sample dendrogram and trait heat map (Figures 1(a) and 1(b)). To achieve a scale-free coexpression network, *β* = 5 was selected during coexpression analysis. To merge highly similar modules, the threshold was set to 0.25. Using the dynamic tree cut method, 32 modules were identified in total (Figures 1(c) and 1(d)). When we constructed module-trait relationships (Figure 2(a)), the tan module indicated a statistically significant value in terms of endothelial cell proliferation function (score = 0.44, *p* = 0.006), while the yellow module had a statistically significant value in terms of endothelial cell migration function (score = 0.41, *p* = 0.01). The tan and yellow module memberships and gene significance for traits were subsequently calculated (Figures 2(b) and 2(c)).

### 3.3. Mining Hub Genes and Key Genes

**Figure 3:**
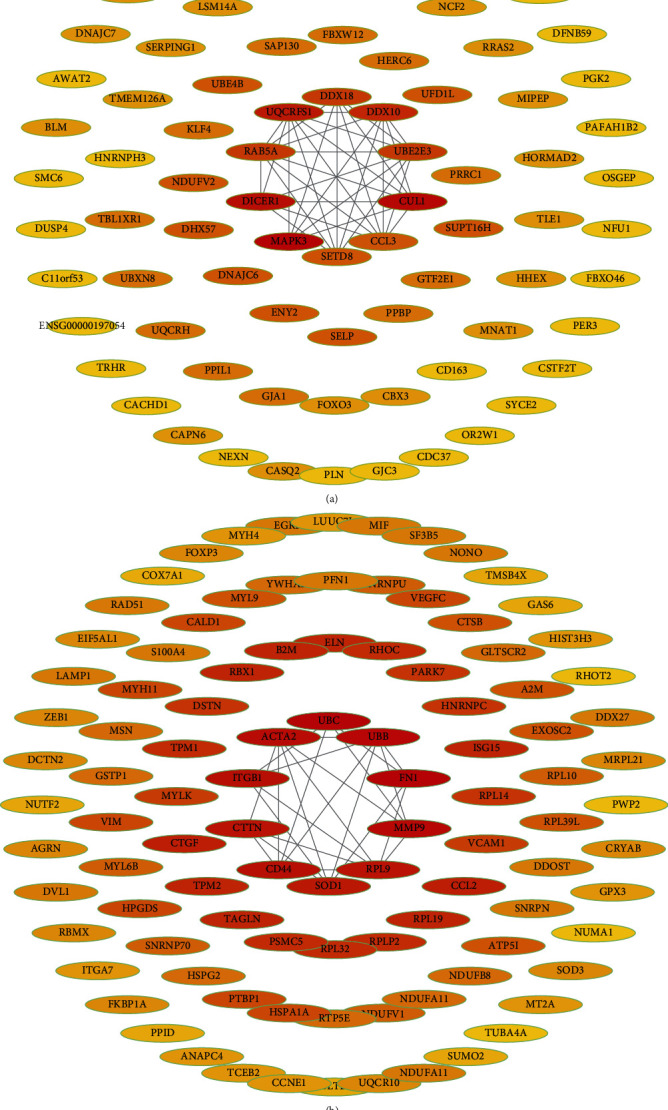
PPI network of genes from the tan and yellow hub modules. (a, b) The higher the number of connected nodes, the darker the color of the node. Ten nodes with the deepest color were selected as the key genes.

**Figure 4:**
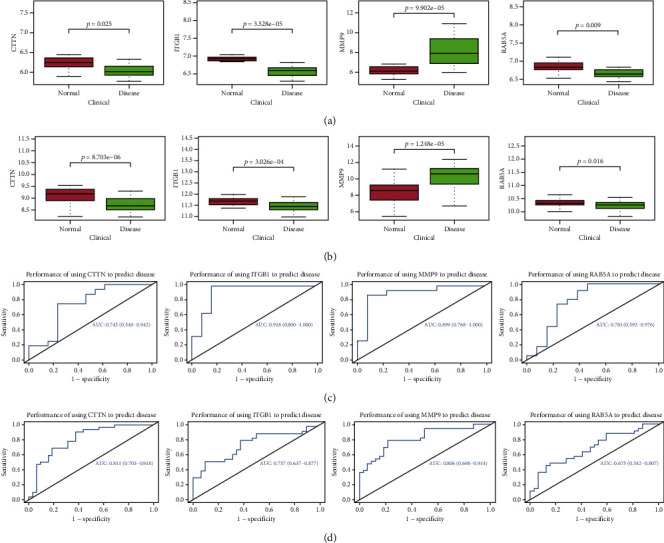
Identification and verification of key genes. (a, b) The comparison of hub gene expression between normal and disease groups. Four key genes (*CTTN*, *ITGB1*, *MMP9*, and RAB5A) were significantly different in the two conditions (GSE28829: *CTTN* (p = 0.025), *ITGB1* (*p* = 3.528*e* − 05), *MMP9* (*p* = 9.902*e* − 05), and *RAB5A* (*p* = 0.009); GSE43292: *CTTN* (*p* = 8.703*e* − 06), *ITGB1* (*p* = 3.026*e* − 04), *MMP9* (*p* = 1.248*e* − 05), and *RAB5A* (*p* = 0.016). (c, d) The ROC curves for *CTTN*, *ITGB1*, *MMP9*, and *RAB5A* demonstrating their ability to predict atherosclerosis (GSE28829 and GSE43292, respectively).

The PPI network of the genes in the two (tan and yellow) modules was obtained (Figures 3(a) and 3(b)), with the top 10 genes selected as hub genes. In total, 10 genes (*MAPK3*, *CUL1*, *DICER1*, *UQCRFS1*, *DDX10*, *RAB5A*, *DDX18*, *UBE2E3*, *PAICS*, and *NDUFV2*) were obtained in tan modules associated with endothelial cell proliferation (Table 2), and 10 genes (*FN1*, *UBC*, *UBB*, *MMP9*, *ACTA2*, *ITGB1*, *CD44*, *RPL9*, *CTTN*, and *SOD1*) were obtained in yellow modules associated with endothelial cell migration (Table 3). The verification of the hub gene showed that in GSE28829 and GSE43292 datasets, the expression of *CTTN* and *ITGB1* (considered as key genes closely related to endothelial cell migration function) from atherosclerotic groups decreased significantly, while the expression of *MMP9* increased(Figures 4(a) and 4(b)). However, *RAB5A* is the only gene associated with endothelial cell proliferation whose expression decreased significantly. The ROC analysis showed that these four key genes can predict atherosclerosis well (Figures 4(c) and 4(d)). The above results suggest that these key genes are related to the ECD in atherosclerosis.

### 3.4. Functional Enrichment Analysis of Genes in Hub Modules

**Figure 5:**
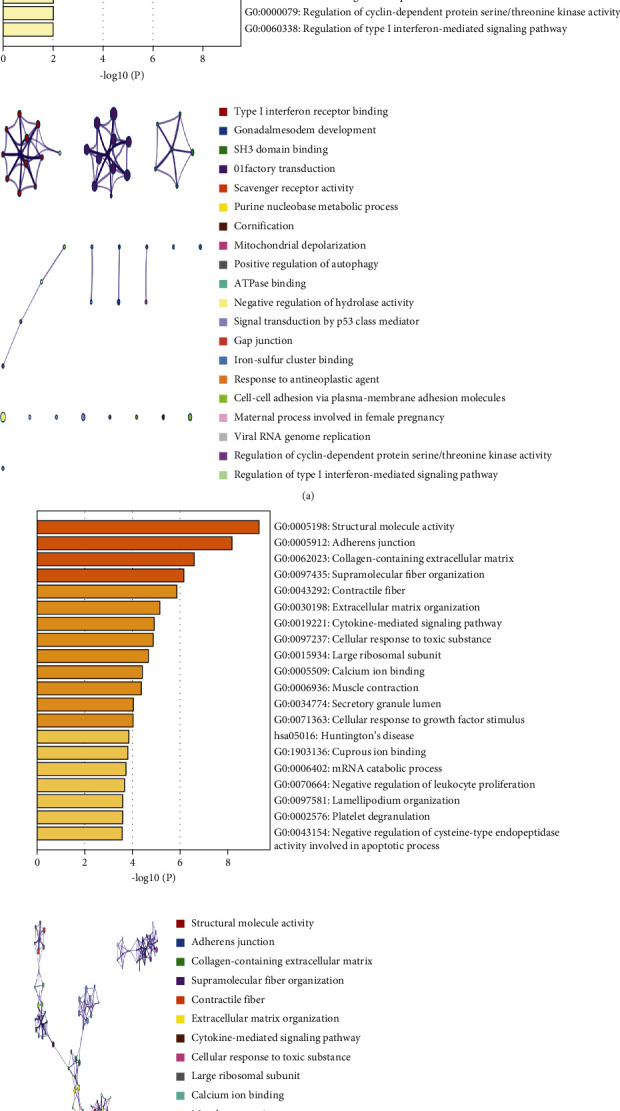
GO and KEGG enrichment analysis of genes in tan and yellow modules. (a, b) The first 20 enriched terms are shown as a bar chart on top. The network diagram on the bottom is constructed with each enrichment term as a node and the similarity of the node as the edge. Nodes with the same cluster ID are with the same color. Min overlap ≥ 3 and *p* ≤ 0.01 were considered to be statistically significant.

The top 20 GO and KEGG terms were extracted and shown. There was a significant difference in the enriched terms and degrees (Figures 5(a) and 5(b)). Tan module genes were mainly enriched for GO:0005132: type I interferon receptor binding, GO:0007506: gonadal mesoderm development, GO:0017124: SH3 domain binding, and hsa04740: olfactory transduction. Genes in the yellow module were mainly enriched for GO:0005198: structural molecule activity, GO:0005192: adherens junctions, GO:0062023: collagen-containing extracellular matrix, GO:0097435: supramolecular fiber organization, and GO:0043292: contractile fibers. Notably, the transcriptional regulatory network of all genes in the hub module was constructed to provide reference for subsequent experiments (Supplemental File Fig. 1A-B).

### 3.5. Disease Correlation Analysis and GSVA

**Figure 6:**
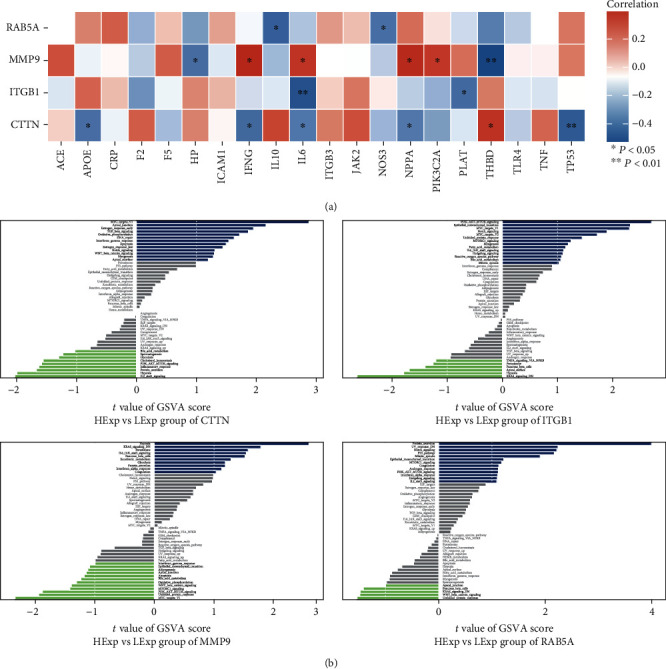
The GSVA and disease association of key genes. (a) Each row corresponds to a key gene. Cells are color-coded according to the legend. Cells with statistically significant *p* values are shown as ∗ or ∗∗. (b) The vertical axis represents the change in signaling pathways in the corresponding samples with low or high key gene expression. The horizontal axis represents the degree of variation of each pathway in the gene set. The green bar represents the sample with high expression in the key gene, while the blue bar shows the opposite.

The disease correlation analysis (Figure 6(a)) showed that the expression of *CTTN* was positively correlated with the THBD, while it was negatively correlated with APOE, IFNG, IL6, NPPA, and TP53 (*p* < 0.01). The expression of *ITGB1* was negatively correlated with the IL-6 (*p* < 0.01) and PLAT (*p* < 0.05). The expression of *MMP9* was positively correlated with IFNG, IL6, NPPA, and PIK3C2A (*p* < 0.05) but negatively correlated with HP (*p* < 0.05) and THBD (*p* < 0.01). The expression of *RAB5A* was negatively correlated with IL-10 and NOS3 (*p* < 0.05).

The results of GSVA (Figure 6(b)) show that when *CTTN* was lowly expressed, the Notch signaling, interferon *γ* response, WNT *β*-catenin signaling, MYC targets V1, and TGF*β* signaling were enriched. The Notch signaling, MYC targetsV, PI3K-AKT-MTOR signaling, and IL6-JAK-STAT3 signaling were enriched when *ITGB1* was lowly expressed. The Notch signaling, MTORC1 signaling, P53 pathway, PI3K-AKT-MTOR signaling, and interferon *α* response were enriched in the *RAB5A* low-expression group while PI3K-AKT-MTOR signaling, interferon *α* response, MYC targetsV, WNT *β*-catenin signaling, and MTORC1 signaling were enriched in the *MMP9* high-expression group.

### 3.6. Results of Verification of Key Gene Expression

**Figure 7:**
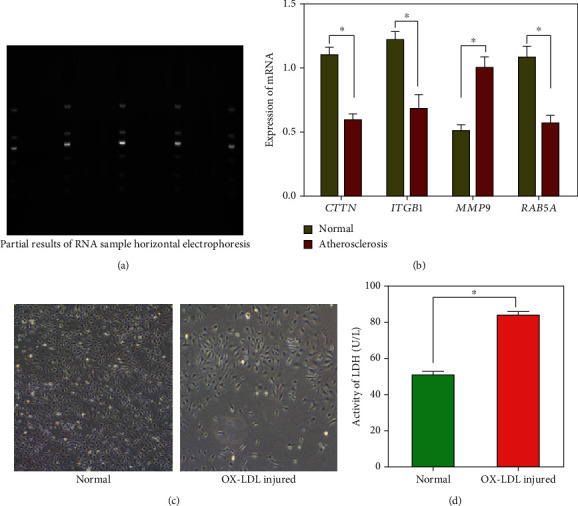
(a) Some results of horizontal electrophoresis of RNA samples from clinical specimens are shown. (b) The Q-PCR results of key genes; *CTTN*, *ITGB1*, and *RAB5A* in atherosclerotic samples were significantly lower than those in the normal group (*p* < 0.05). On the contrary, *MMP9* in atherosclerotic samples was higher than that in the control group (*p* < 0.05). (c) Establishment of the injured HCAEC model. In the normal group, the HCAECs were uniform and regular, spindle-shaped, and closely connected with each other. In the injured group, the HCAEC morphology was nonuniform, the connection between cells was not very close, and the arrangement was irregular. (d) The LDH activity assays showed that compared to the normal group, LDH activity was significantly increased in the ox-LDL-injured group (*p* < 0.05). The above results show that the cell model is successful.

The total RNAs of 16 atherosclerotic and normal aortic wall samples were successfully obtained and passed the quality evaluation. Some results of horizontal electrophoresis of RNA samples are shown in Figure 7(a). It can be seen from the Q-PCR results (Figure 7(b)) that *CTTN*, *ITGB1*, and *RAB5A* in atherosclerotic samples were significantly lower than those in the normal group (*p* < 0.05). On the contrary, *MMP9* in atherosclerotic samples was higher than that in the normal group (*p* < 0.05). This is consistent with the results of bioinformatics analysis.

### 3.7. Establishment of the Injured HCAEC Model and *RAB5A* Overexpression and Knockdown Model *In Vitro*

In the normal group, the HCAECs were uniform and regular, spindle-shaped, and closely connected with each other. In the injured group, the HCAEC morphology was nonuniform, the connection between cells was not very close, and the arrangement was irregular (Figure 7(c)). Compared with the normal cells, the LDH activity in the injured group was significantly increased (*p* < 0.05) (Figure 7(d)). The above results show that the cell model of injured HCAECs is successful.

**Figure 8:**
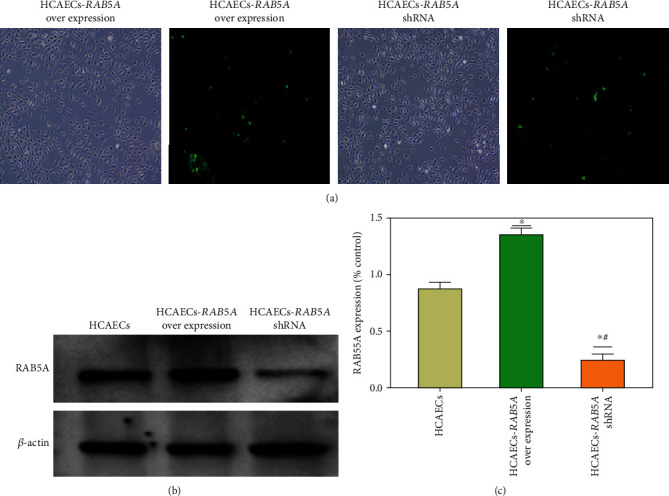
(a) RAB5A overexpression and shRNA plasmid transfection HCAECs in vitro. There was no significant difference in transfection efficiency between two groups (28.7% and 27.3%), and the green fluorescence carried by the plasmid itself can be seen. (b, c) The results of measuring the expression of RAB5A protein after transfection are shown in (b). Relative quantitative comparison of western blot analyses in (C). ^∗^*p* < 0.05, compared to the HCAEC group; ^#^*p* < 0.05, compared to the HCAEC-RAB5A overexpression group.

As shown in Figure 8(a), the transfection efficiency of the RAB5A overexpression and knockdown group is similar, and the green fluorescence carried by the plasmid itself can be seen. The expression results of *RAB5A* protein after transfection are shown in Figures 8(b) and 8(c). The expression of RAB5A in the overexpression group was significantly higher than that in the other two groups (*p* < 0.05). At the same time, the expression of RAB5A in the knockdown group was significantly lower than that in the normal group (*p* < 0.05).

### 3.8. The Effect of *RAB5A* on ox-LDL-Injured HCAEC Function of Proliferation

**Figure 9:**
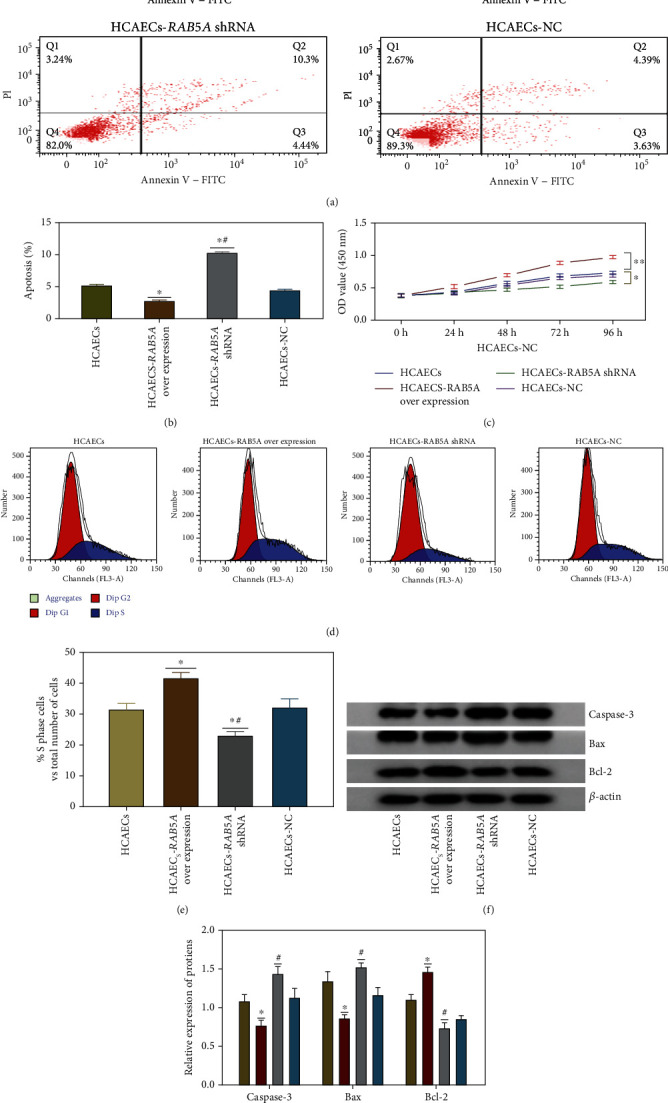
(a) The endothelial cell apoptosis rates in the HCAEC, HCAEC-RAB5A overexpression, HCAEC-RAB5A shRNA, and HCAEC-NC groups were 5.18%, 2.75%, 10.3%, and 4.39%, respectively. (b) Column analysis chart of the apoptosis rate in each group. ^∗^*p* < 0.05, compared to the HCAEC group; ^#^*p* < 0.05, compared to the HCAEC-RAB5A overexpression group. (c) The cell proliferation of each group was compared at each time point (24 h, 48 h, 72 h, and 96 h). ^∗∗^*p* < 0.05, HCAEC-RAB5A overexpression compared to HCAEC group; ^∗^*p* < 0.05, HCAEC-RAB5A shRNA compared to HCAEC group. (d, e) The cell cycle analysis for all groups. (d) The cell cycle analysis results are shown in (d). The area under the red curve represents the G1 phase of the cell cycle. The area under the curve filled with slashes represents the S phase of the cell cycle. (e) Column analysis chart of the cell cycle in all groups. (f, g) Apoptosis-related protein expressions for all groups. (f) The expression levels of Bax, caspase-3, and Bcl-2 proteins in each group. (g) Relative quantitative comparison of western blot analyses.

The cell apoptosis rates in the HCAEC, HCAEC-RAB5A overexpression, HCAEC-RAB5A shRNA, and HCAEC-NC groups were 5.18%, 2.75%, 10.3%, and 4.39%, respectively (Figure 9(a)). Compared with the HCAEC group, the cell apoptosis rates decreased in the HCAEC-RAB5Aoverexpression group but increased in the HCAEC-RAB5A shRNA group (*p* < 0.05). There was no significant difference in the cell apoptosis rate between the HCAECs and HCAECs-NC (*p* > 0.05). A column analysis chart of the apoptosis rate is shown in Figure 9(b).

The results of the CKK8 test showed that as shown in Figure 9(c), the OD value in each group reflecting the cell proliferation had no significant difference at 24 h. At 48 h, the cell growth rate in each group was different (*p* > 0.05). At 72 h and 96 h, the results showed that the proliferation activity of the HCAEC-RAB5A shRNA group decreased lower than that in the other three groups (*p* < 0.05). When RAB5A was overexpressed, the cell growth rate was fastest compared to that of other groups (*p* < 0.01). There was no significant difference in OD between the normal group and the NC group (*p* > 0.05). This experiment proved that overexpression of RAB5A could improve the proliferation of injured HCAECs. When the expression of RAB5A decreased significantly, this function disappeared.

The cell cycle analysis results are shown in Figures 9(d) and 9(e). The S phase proportion of cells in the five groups was 31.31%, 41.91%, 23.62%, and 32.41, respectively. This result is consistent with the CKK8 test. When RAB5A was overexpressed, more cells were in the S phase of the cell cycle (*p* < 0.05). On the contrary, when RAB5A expression was knocked down, the HCAEC-RAB5A shRNA group had the least cell number in the S phase (*p* < 0.05).

After 48 h of transfection and treatment, there were no significant differences in apoptosis-related protein expressions between HCAEC and HCAEC-NC groups (*p* > 0.05) (Figure 9(f)). Compared to the HCAEC-RAB5A overexpression group, Bax and caspase-3 proteins were upregulated, but Bcl-2 protein was downregulated in HCAECs-RAB5A shRNA (*p* < 0.05). Compared with the HCAEC group, Bax and caspase-3 proteins were downregulated, whereas Bcl-2 protein was upregulated in the HCAEC-RAB5A overexpression group (*p* < 0.05). Relative quantitative comparison of western blot analyses is shown in Figure 9(g).

### 3.9. Results of the Effect of *RAB5A* on ox-LDL-Injured HCAEC Migration Function

**Figure 10:**
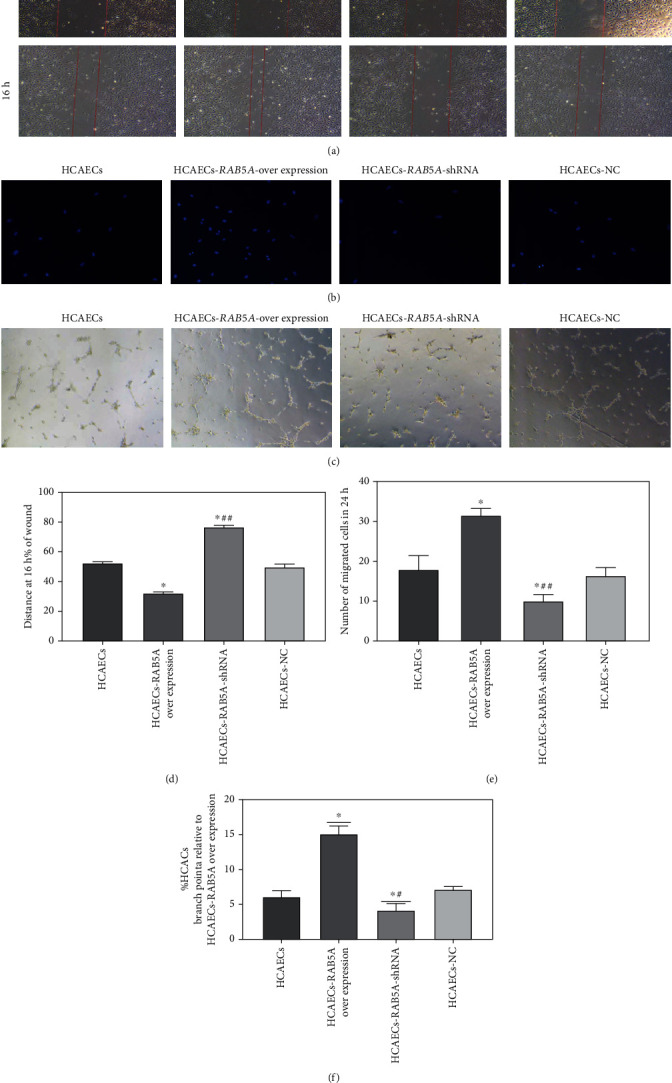
(a, d) Wound healing assay in all groups. (a) Migration distance of HCAECs in different conditions. (d) Relative quantitative comparison of healing speed analyses mentioned in (a). (b, e) Transwell migration assay in all groups. (b) Representative photomicrographs showing migrating cells in different conditions. (e) Quantification of number of migrating cells demonstrated that overexpression of RAB5A promoted HCAEC migration function (*n* = 6/group). (c, f) Tube formation assay. (c) Representative photomicrographs showing tube formation of HCAECs in different conditions. (f) Quantification of HCAEC tube junction numbers demonstrated that overexpression of RAB5A ameliorated HCAECs to form capillary-like structures (*n* = 6/group). ^∗^*p* < 0.05, compared to the HCAEC group; ^#^*p* < 0.05, compared to the HCAEC-RAB5A overexpression group.

The results of the wound healing assay (Figures 10(a) and 10(c)) showed that at 16 hours after the beginning of the experiment, the migration distance of the HCAEC-RAB5A overexpression group was longer than that of the HCAEC group (*p* < 0.05), and the migration distance of the HCAEC-RAB5A shRNA group was shorter than that of HCAEC-RAB5A overexpression and HCAEC groups (*p* < 0.05).

After 24 hours of the Transwell migration assay, the migration number of cells in each group was determined by DAPI staining. It can be seen that the migration number of cells in the HCAEC-RAB5A overexpression group was higher than that in HCAEC and HCAEC-RAB5A shRNA groups (Figures 10(b) and 10(d); *p* < 0.05). The migration number of cells in the HCAEC-RAB5A shRNA group was the least among each group.

Furthermore, the tube formation assay was performed to confirm that the increased expression of RAB5A can promote angiogenesis of HCAECs. Compared to the HCAEC and HCAEC-RAB5A shRNA groups, the HCAEC-RAB5A overexpression group promoted HCAECs to form capillary-like structures (Figures 10(c) and 10(e)).

## 4. Discussion

According to the WHO, cardiovascular disease is still one of the leading causes of mortality globally. During the progression of atherosclerosis, endothelial cells (ECs) display abnormal proliferation and migration that is responsible for the destruction of the integrity of the endothelium [15]. This consequently aggravates lipid deposition and fibrous cap rupture [16]. Participating in atherogenesis, anomalous ECs produce multiple cytokines [17]. These include interleukins, adhesion molecules, and matrix metalloproteinases. ECD is considered to precede atherosclerosis and induces abnormal proliferation and migration of ECs recognized in the early stages of atherosclerosis [18]. Some experiments aimed at understanding the role of ECD in the development of atherosclerosis have been conducted. Zhang et al. [19] found that circ_0003204 RNA inhibits proliferation and migration of endothelial cells in atherosclerosis via the miR-370-3p/TGF*β*R2/phosph-SMAD3 axis, while Peng et al. [20] declared that thymic stromal lymphopoietin promotes endothelial cell proliferation and migration in atherosclerosis by inducing HOTAIR activation. Nevertheless, the knowledge surrounding ECD and genes involved in atherosclerosis remains elusive. Studying the unknown genetic mechanisms underlying ECD in atherosclerosis could provide us with potential targets for reversing and ameliorating atherosclerosis. Because of the lack of clinical characteristic data of endothelial cell dysfunction, it is difficult to correlate and analyze the changes in gene expression with the changed cell function in the past. The emergence of ssGSEA, WGCNA, and Molecular Signatures Database makes this attempt possible.

The relationship between atherosclerosis-associated EC eigengenes and clinical features was revealed by ssGSEA and WGCNA in this paper (Figures 1–3). This is the first attempt to reveal the internal relationship between endothelial cell function and genes, but this method has been successful in studying tumor immune cell infiltration. Among these analysis results, functional enrichment analysis indicates the possible enrichment pathways involved in the tan and yellow modules, respectively (Figure 5). Structural molecule activity and adherens junction were enriched in the yellow module. Increased adhesion of monocytes to endothelial cells may be considered an early symptom of atherosclerosis [21]. Endothelial cells appear to respond to different stimuli by expressing adhesion molecules, thereby increasing the adhesion of monocytes and T lymphocytes to endothelial cells [21]. Type I interferon receptor binding and regulation of the type I interferon-mediated signaling pathway were enriched in the tan module associated with endothelial cell proliferation.

This is consistent with findings that IFN-*γ* activates T cells, macrophages, endothelial cells, and fibroblasts that give rise to the formation of atherosclerotic aneurysms in the abdominal aorta [22]. Results of another study show that INF-*γ* causes the expression of NOS, which exerts a wide variety of effects on the vessel walls via *in vivo* production of NO [23]. Such analysis results let us know which possible participants are involved in the changes of endothelial cell migration and proliferation. Obviously, this requires further research and analysis.

Further research results show that the *RAB5A*, *CTTN*, *ITGB1*1and *MMP9* can be used to predict atherosclerosis which is verified by GSE28829 and GSE43292 (Figure 4). Subsequently, we verified the expression of these genes in the normal and atherosclerotic aortic walls (Figures 7(a) and 7(b)). Fortunately, the performance of key genes is satisfactory. Due to the characteristics of multiple etiologies and mechanisms of atherosclerosis, it is certain that many other important components may be involved, such as some proteins, transcription factors, and cytokines [24]. Therefore, we calculated the relationship between these genes and known markers of atherosclerosis, and we also analyzed the signal pathways that may be involved in the abnormal expression of these genes leading to disease using GSVA. As shown in Figure 6(a), key genes are associated with some known atherosclerosis markers. For example, *RAB5A* was correlated with IL-10 and NOS3. The GSVA result showed (Figure 6(b)) that when the expression of *RAB5A*, *CTTN*, and *ITGB1* decreased and the expression of *MMP9* increased, Notch signaling, PI3K-Akt signaling, MYC targets V, interferon *α*/*γ* response, etc. are involved. Of course, each gene also has specific signal transduction pathways involved. For instance, NOS3, IL-10, and P53 pathway are related to *RAB5A*. The above results suggest that these genes may be involved in atherosclerosis through these markers and/or signal pathways. This also provides us with research directions.

*RAB5A* belongs to the Rab-GTPase family of proteins, plays an important role in endocytosis, exocytosis, and vesicle transport, and has been implicated in a large range of diseases ranging from cancer to bacterial and viral infections [25]. In the previous studies, there have been some studies on the function of RAB5A. For example, *RAB5A* advances the migration and invasion of hepatocellular carcinoma through upregulating Cdc42 [26] (Yang X et al., 2018). *RAB5A* is overexpressed in oral cancer and promotes invasion through ERK/MMP signaling [27]. Moreover, *RAB5A*-mediated autophagy can regulate the phenotype transition and cell behavior of VSMCs through the activation of the ERK1/2 signaling pathway [28]. The function of *RAB5A* mainly involves endocytosis, exocytosis, and vesicle transport, which can play an important role in the occurrence and development of atherosclerosis [29, 30]. *RAB5A* functions and potential relationships with atherosclerosis have aroused our strong interest. So, we established the cell model of atherosclerosis and detected the changes in endothelial cell function caused by overexpression or inhibition of RAB5A expression.

Because *RAB5A* is located in the tan module related to endothelial cell proliferation, firstly, we analyzed the effect of *RAB5A* on endothelial cell proliferation. The flow cytometry results of cell apoptosis showed that compared with the HCAEC and HCAEC-RAB5A shRNA groups, endothelial cells in the HCAEC-RAB5A overexpression group had the lowest apoptosis rate (Figures 9(a) and 9(b)) which was consistent with the expression trend of apoptosis-related proteins (Figures 9(f) and 9(g)). Endothelial cells in the HCAEC-RAB5A overexpression group had less caspase-3 and Bax and more Bcl-2 expression. From another perspective, we can see that, when *RAB5A* is overexpressed, endothelial cells have higher cell proliferation efficiency (Figure 9(c)). The cell cycle assay also confirmed that more cells were in the S phase of the cell cycle when RAB5A was overexpressed (Figures 9(d) and 9(e)). So, we speculated that *RAB5A* can affect atherosclerosis by regulating the proliferation of endothelial cells. However, this effect occurs at least 48 hours after transfection. As mentioned before, it was found that *RAB5A* has a strongly negative correlation with IL-10 and NOS3 (eNOS). The evidence that IL-10 and NOS3 play a positive role in inhibiting the development of atherosclerosis has been discussed elsewhere [31, 32]. Moreover, GSVA analysis demonstrated that low *RAB5A* expression is primarily associated with interferon *α*/*γ* response, NOS3, IL-10, PI3K-AKT, and Notch signaling pathways.

Enriched in tan module genes associated with endothelial cell proliferation in this study, some experiments have confirmed the role of interferon and related signaling pathways in the pathogenesis of atherosclerosis. For example, Li et al. [33] concluded that IFN-*α* can increase the uptake of ox-LDL and enhance foam cell formation by upregulating macrophage SR class A expression. This is achieved via enhancing its promoter activities, with the PI3K/Akt signaling pathway appearing to be involved in this process. Another study suggests that excessive expression of IFN-I through the activation of TLR7/9 signaling may induce accelerated atherosclerosis through the depletion or dysfunction of endothelial progenitor cells [34]. Many studies have also confirmed that interferon *α*/*γ* response is closely related to cell cycle and cell proliferation. For example, IFN-*γ* modulates the self-renewal, cell cycle entry, and proliferation of HSCs [35]. IFN-*γ* inhibits endothelial cell proliferation, migration, and reendothelialization of injured arteries by inhibiting the nuclear factor-kappa B pathway [36]. Gomez and Reich [37] provide evidence that IFN can stimulate the proliferation of primary human endothelial cells by an increase in DNA synthesis assessed with thymidine incorporation, an increase in G and M cell cycle phases assessed with flow cytometric analysis, and an increase in the cell number. The Rab family is also closely related to the IFN family in some cell functions (*RAB5A* and IFN-*γ*). The interaction mainly involves the selective effects of synthesis, processing, and nucleotide exchange, which can enhance the membrane transport function and further enhance some cell functions [38]. Targeting *RAB5A* through IFN might therefore have potential therapeutic effects on atherosclerosis. Notch signaling has also been shown to play a variety of roles in atherosclerosis. Lin et al. [39] reported that the activation of the Notch signaling pathway can induce vascular endothelial dysfunction and promote the development of atherosclerotic lesions. Moreover, Notch signaling promotes endothelial cell proliferation and vessel growth in postnatal long bone [40]. *RAB5A* cooperates with synthetic exosome-like nanoparticles to inhibit Notch signaling and then affect the proliferation of human pancreatic tumor soj-6 cells [41]. These above findings support our conclusion that *RAB5A* may play an important role in the development of atherosclerosis by affecting the proliferation function of endothelial cells. It is important that the interferon response and Notch signaling pathway are likely to be involved in this process.

Previous researchers found that *RAB5A* can accelerate the progress of cancer by affecting the migration of cancer cells [42]. This shows the diversity of *RAB5A* functions, so *RAB5A* may also promote the development of atherosclerosis by affecting the migration function of endothelial cells.

Our experimental results show that compared with the HCAEC and HCAEC-RAB5A shRNA groups, endothelial cells in the HCAEC-RAB5A overexpression group had stronger migration ability (Figures 10(a) and 10(b)). Moreover, overexpression of RAB5A alleviates the impaired vessel tube formation capacity of endothelial cells caused by ox-LDL (Figure 10(c)). It is well characterized that vascular endothelial growth factor A and its receptor (VEGFR2) are important regulators of vascular physiology. *RAB5A* regulation of VEGFR2 trafficking and signaling linked to endothelial cell migration in the early endosome sorting of both quiescent and activated VEGFR2 has been demonstrated in a recent study [43, 44]. Similarly, another study has found that *RAB5A* knockdown inhibits LPS-induced endothelial barrier dysfunction *in vivo* and that *RAB5A* may be a potential therapeutic target for preventing endothelial barrier disruption and vascular inflammation by regulating VE-cadherin internalization [45]. What is more, VE-cadherin is closely related to cell migration [46, 47]. Therefore, we speculate that *RAB5A* may affect the migration function of endothelial cells by affecting VEGFR2 and VE-cadherin.

The changes in proliferation and migration functions of ECs have been implicated as being a critical step in the progression of atherosclerosis [48, 49]. Therefore, the genes related to the functional changes of endothelial cells have become a research hotspot. Based on ssGSEA and WGCNA, hub modules and key genes were identified. The *RAB5A*, *CTTN*, *ITGB1*, and *MMP9* key genes may act as potential targets for medical therapy and diagnostic biomarkers. In particular, when *RAB5A* is overexpressed in ox-LDL-injured endothelial cells, endothelial cells will have better proliferation and migration ability and reduce the occurrence of endothelial cell dysfunction to a certain extent. So, *RAB5A* may be involved in the pathogenesis of atherosclerosis by changing the proliferation and migration function of endothelial cells through some cytokines and/or signaling pathways, such as the interferon response, Notch signaling pathways, VEGFR2, VE-cadherin, NOS3, and IL-10. Further studies are needed to elucidate the detailed biological function of these genes associated with ECD in the pathogenesis of atherosclerosis.

## Figures and Tables

**Table 1:** The primer sequences of key genes.

Gene name	Forward primer	Reverse primer
RAB5A	CAAGAACGATACCATAGCCTAGCAC	CTTGCCTCTGAAGTTCTTTAACCC
CTTN	GCTTTGAGTATCAAGGCAAAACG	CCAAGGGCACATTTGTCTTGT
ITGB1	CCTACTTCTGCACGATGTGATG	CCTTTGCTACGGTTGGTTACATT
MMP9	GGGACGCAGACATCGTCATC	TCGTCATCGTCGAAATGGGC
*β*-Actin	CCTGTACGCCAACACAGTGC	ATACTCCTGCTTGCTGATCC

**Table 2:** The hub genes of the tan modules.

Gene	Gene accession
UQCRFS1	ENSP00000306397
PAICS	ENSP00000382595
NDUFV2	ENSP00000327268
DDX10	ENSP00000314348
MAPK3	ENSP00000263025
RAB5A	ENSP00000273047
DICER1	ENSP00000437256
CUL1	ENSP00000326804
DDX18	ENSP00000263239
UBE2E3	ENSP00000386788

**Table 3:** The hub genes of the yellow modules.

Gene	Gene accession
FN1	ENSP00000346839
RPL9	ENSP00000400467
UBC	ENSP00000441543
MMP9	ENSP00000361405
ACTA2	ENSP00000402373
ITGB1	ENSP00000379350
CD44	ENSP00000398632
SOD1	ENSP00000270142
CTTN	ENSP00000365745
UBB	ENSP00000304697

## Data Availability

The datasets used and analyzed during the current study are available from the corresponding author on reasonable request.

## Supplementary Materials

Supplementary 1 Gene expression matrix of the GSE83500 dataset: firstly, download a series matrix file of GSE83500 from the NCBI GEO public database. Secondly, the raw data was used to transform microarray information into gene expression data through the following process: (1) probes matching several genes were eliminated, as were genes with a negative expression value, and (2) the average value was used as the expression data for genes matching more than a probe.

Supplementary 2 Quantitative results of endothelial cell function: first, the dataset of endothelial cell migration proliferation was predefined by the Molecular Signatures Database (v7.0). Then, the migration/proliferation of endothelial cells in each sample was quantified by the ssGSEA algorithm. Each value represents the quantified score.

Supplementary 3 Module membership and gene significance for a trait: this Excel table contains the names of 10,000 genes and 32 modules and GS/p-GS (gene significance) and MM/p-MM (module membership) values.

Supplementary 4 After WGCNA, a total of 283 genes were included in the tan module.

Supplementary 5 After WGCNA, a total of genes were included in the yellow module.

Supplementary 6 Construction of the transcriptional regulatory network of all genes in the hub module (A-B). (A) The transcriptional regulatory network of the tan module. (B) The transcriptional regulatory network of the yellow module.

Supplementary 7 Flow chart of this experiment.

Supplementary 8 The protocol of Q-PCR in this study.

Supplementary 9 The instructions of Lipofectamine® 2000 Reagent (Invitrogen, USA).

Supplementary 10 Short description of GSE83500\GSE28829\GSE43292 datasets.

## References

[1] Hartiala J., Schwartzman W. S., Gabbay J., Ghazalpour A., Bennett B. J., Allayee H. (2017). The Genetic Architecture of Coronary Artery Disease: Current Knowledge and Future Opportunities. *Current atherosclerosis reports.*.

[2] Said M. A., Verweij N., van der Harst P. (2018). Associations of Combined Genetic and Lifestyle Risks With Incident Cardiovascular Disease and Diabetes in the UK Biobank Study. *JAMA cardiology.*.

[3] Cahill P. A., Redmond E. M. (2016). Vascular endothelium - Gatekeeper of vessel health. *Atherosclerosis.*.

[4] Gimbrone M. A., Garcia-Cardena G. (2016). Endothelial Cell Dysfunction and the Pathobiology of Atherosclerosis. *Circ Res.*.

[5] Stary H. C. (2000). Natural history and histological classification of atherosclerotic lesions: an update. *Arteriosclerosis, Thrombosis, and Vascular Biology*.

[6] Cooke J. P. (2000). Does ADMA cause endothelial dysfunction. *Arterioscler Thromb VascBiol.*.

[7] Kitamoto S., Egashira K. (2004). Endothelial dysfunction and coronary atherosclerosis. *Current Drug Targets - Cardiovascular & Haematological Disorders.*.

[8] Zhang Y., Chen N., Zhang J., Tong Y. (2013). Hsa-let-7g miRNA targets caspase-3 and inhibits the apoptosis induced by ox-LDL in endothelial cells. *International journal of molecular sciences.*.

[9] Zhang S., Zhu X., Li G. (2020). E2F1/SNHG7/miR-186-5p/MMP2 axis modulates the proliferation and migration of vascular endothelial cell in atherosclerosis. *Life Sciences*.

[10] Ye L., Zhang T., Kang Z. (2019). Tumor-Infiltrating Immune Cells Act as a Marker for Prognosis in Colorectal Cancer. *Front Immunol.*.

[11] Langfelder P., Horvath S. (2008). WGCNA: an R package for weighted correlation network analysis. *BMC Bioinformatics.*.

[12] Lin X., Li J., Zhao Q., Feng J. R., Gao Q., Nie J. Y. (2018). WGCNA Reveals Key Roles of IL8 and MMP-9 in Progression of Involvement Area in Colon of Patients with Ulcerative Colitis. *Current medical science.*.

[13] Liu Z., Li M., Fang X. (2019). Identification of surrogate prognostic biomarkers for allergic asthma in nasal epithelial brushing samples by WGCNA. *J Cell Biochem.*.

[14] Giulietti M., Occhipinti G., Principato G., Piva F. (2016). Weighted gene co-expression network analysis reveals key genes involved in pancreatic ductal adenocarcinoma development. *Cell Oncol (Dordr).*.

[15] Badimon L., Vilahur G. (2014). Thrombosis formation on atherosclerotic lesions and plaque rupture. *J Intern Med.*.

[16] Qiu J., Wang G., Zheng Y., Hu J., Peng Q., Yin T. (2011). Coordination of Id1 and p53 activation by oxidized LDL regulates endothelial cell proliferation and migration. *Ann Biomed Eng.*.

[17] Tousoulis D., Oikonomou E., Economou E. K., Crea F., Kaski J. C. (2016). Inflammatory cytokines in atherosclerosis: current therapeutic approaches. *Eur Heart J.*.

[18] Peppas N. A., Bures P., Leobandung W. S., Ichikawa H. (2000). Hydrogels in pharmaceutical formulations. *European Journal of Phmarmaceutics and Phmarmaceutics*.

[19] Zhang S., Song G., Yuan J. (2020). RETRACTED ARTICLE: Circular RNA circ_0003204 inhibits proliferation, migration and tube formation of endothelial cell in atherosclerosis via miR-370-3p/TGF*β*R2/phosph-SMAD3 axis. *J Biomed Sci.*.

[20] Peng Y., Meng K., Jiang L. (2017). Thymic stromal lymphopoietin-induced HOTAIR activation promotes endothelial cell proliferation and migration in atherosclerosis. *Biosci Rep.*.

[21] Wang C., Varshney R. R., Wang D. A. (2010). Therapeutic cell delivery and fate control in hydrogels and hydrogel hybrids. *Adv Drug Deliv Rev.*.

[22] Vrana N. E., Liu Y., McGuinness G. B., Cahill P. A. (2008). Characterization of Poly(vinyl alcohol)/Chitosan Hydrogels as Vascular Tissue Engineering Scaffolds. *Macromolecular Symposia.*.

[23] Bidarra S. J., Barrias C. C., Granja P. L. (2014). Injectable alginate hydrogels for cell delivery in tissue engineering. *Acta Biomater*.

[24] Kulbertus H., Lancellotti P. (2012). Atherosclerosis: a complex disease. *Rev Med Liege*.

[25] Stenmark H., Olkkonen V. M. (2001). The Rab GTPase family. *Genome Biol.*.

[26] Yang X., Liu Z., Li Y. (2018). Rab5a promotes the migration and invasion of hepatocellular carcinoma by up-regulating Cdc42. *International Journal of Clinical and Experimental Pathology*.

[27] Zhang D., Lu C., Ai H. (2017). Rab5a is overexpressed in oral cancer and promotes invasion through ERK/MMP signaling. *Mol Med Rep.*.

[28] Tan J. Y., Jia L. Q., Shi W. H., He Q., Zhu L., Yu B. (2016). Rab5a-mediated autophagy regulates the phenotype and behavior of vascular smooth muscle cells. *Mol Med Rep.*.

[29] Couto N. F., Rezende L., Fernandes-Braga W. (2020). OxLDL alterations in endothelial cell membrane dynamics leads to changes in vesicle trafficking and increases cell susceptibility to injury. *Biomembranes.*.

[30] Robinson K. A., Apkarian R. P. (1991). Ultrastructure of coronary arterial endothelium in atherosclerotic swine suggests lipid retro-endocytosis. *Scanning Microscopy*.

[31] Kobiyama K., Ley K. Atherosclerosis. *Circulation Research*.

[32] Spartalis M., Spartalis E., Athanasiou A. (2020). The Role of the Endothelium in Premature Atherosclerosis: Molecular Mechanisms. *Current Medicinal Chemistry*.

[33] Li J., Fu Q., Cui H. (2011). Interferon-*α* priming promotes lipid uptake and macrophage-derived foam cell formation: a novel link between interferon-*α* and atherosclerosis in lupus. *Arthritis Rheum.*.

[34] Geng L., Wang S., Li X. (2018). Association between Type I interferon and depletion and dysfunction of endothelial progenitor cells in C57BL/6 mice deficient in both apolipoprotein E and Fas ligand. *Current research in translational medicine*.

[35] Qin Y., Zhang C. (2017). The Regulatory Role of IFN-*γ* on the Proliferation and Differentiation of Hematopoietic Stem and Progenitor Cells. *Stem cell reviews and reports.*.

[36] Jian D., Wang W., Zhou X. (2018). Interferon-induced protein 35 inhibits endothelial cell proliferation, migration and re-endothelialization of injured arteries by inhibiting the nuclear factor-kappa B pathway. *Acta physiologica.*.

[37] Gomez D., Reich N. C. (2003). Stimulation of primary human endothelial cell proliferation by IFN. *Journal of Immunology*.

[38] Alvarez-Dominguez C., Stahl P. D. (1998). Interferon-*γ* Selectively Induces Rab5a Synthesis and Processing in Mononuclear Cells. *THE JOURNAL OF BIOLOGICAL CHEMISTRY.*.

[39] Lin Q. Q., Zhao J., Zheng C. G., Chun J. (2018). Roles of notch signaling pathway and endothelial-mesenchymal transition in vascular endothelial dysfunction and atherosclerosis. *Eur Rev Med Pharmacol Sci.*.

[40] Ramasamy S. K., Kusumbe A. P., Wang L., Adams R. H. (2014). Endothelial Notch activity promotes angiogenesis and osteogenesis in bone. *Nature.*.

[41] Beloribi S., Ristorcelli E., Breuzard G. (2012). Exosomal lipids impact notch signaling and induce death of human pancreatic tumoral SOJ-6 cells. *PLoS One.*.

[42] Pan Y., Wang R., Zhang F. (2015). MicroRNA-130a inhibits cell proliferation, invasion and migration in human breast cancer by targeting the RAB5A. *Int J Clin Exp Pathol.*.

[43] Conigliaro A., Costa V., Lo Dico A. (2015). CD90+ liver cancer cells modulate endothelial cell phenotype through the release of exosomes containing H19 lncRNA. *Molecular cancer.*.

[44] Jopling H. M., Odell A. F., Hooper N. M., Zachary I. C., Walker J. H., Ponnambalam S. (2009). Rab GTPase regulation of VEGFR2 trafficking and signaling in endothelial cells. *Arterioscler Thromb Vasc Biol.*.

[45] Yang J., Yao W., Qian G., Wei Z., Wu G., Wang G. (2015). Rab5-mediated VE-cadherin internalization regulates the barrier function of the lung microvascular endothelium. *Cellular and Molecular Life Sciences*.

[46] Cao J., Ehling M., März S. (2017). Polarized actin and VE-cadherin dynamics regulate junctional remodelling and cell migration during sprouting angiogenesis. *Nature communications.*.

[47] Malinova T. S., Angulo-Urarte A., Nüchel J. (2021). A junctional PACSIN2/EHD4/MICAL-L1 complex coordinates VE-cadherin trafficking for endothelial migration and angiogenesis. *Nature communications.*.

[48] Libby P., Ridker P. M., Hansson G. K., Leducq Transatlantic Network on Atherothrombosis (2009). Inflammation in atherosclerosis: from pathophysiology to practice. *Journal of the American College of Cardiology.*.

[49] Rautou P. E., Leroyer A. S., Ramkhelawon B. (2011). Microparticles from human atherosclerotic plaques promote endothelial ICAM-1-dependent monocyte adhesion and transendothelial migration. *Circ Res.*.

