# Supplementary figures and images for: Identification of Key Genes Associated with Endothelial Cell Dysfunction in Atherosclerosis Using Multiple Bioinformatics Tools
Source: Biomed Res Int. 2022 Jan 10;2022:5544276. doi: 10.1155/2022/5544276 (PMC8764276; doi:10.1155/2022/5544276)

A

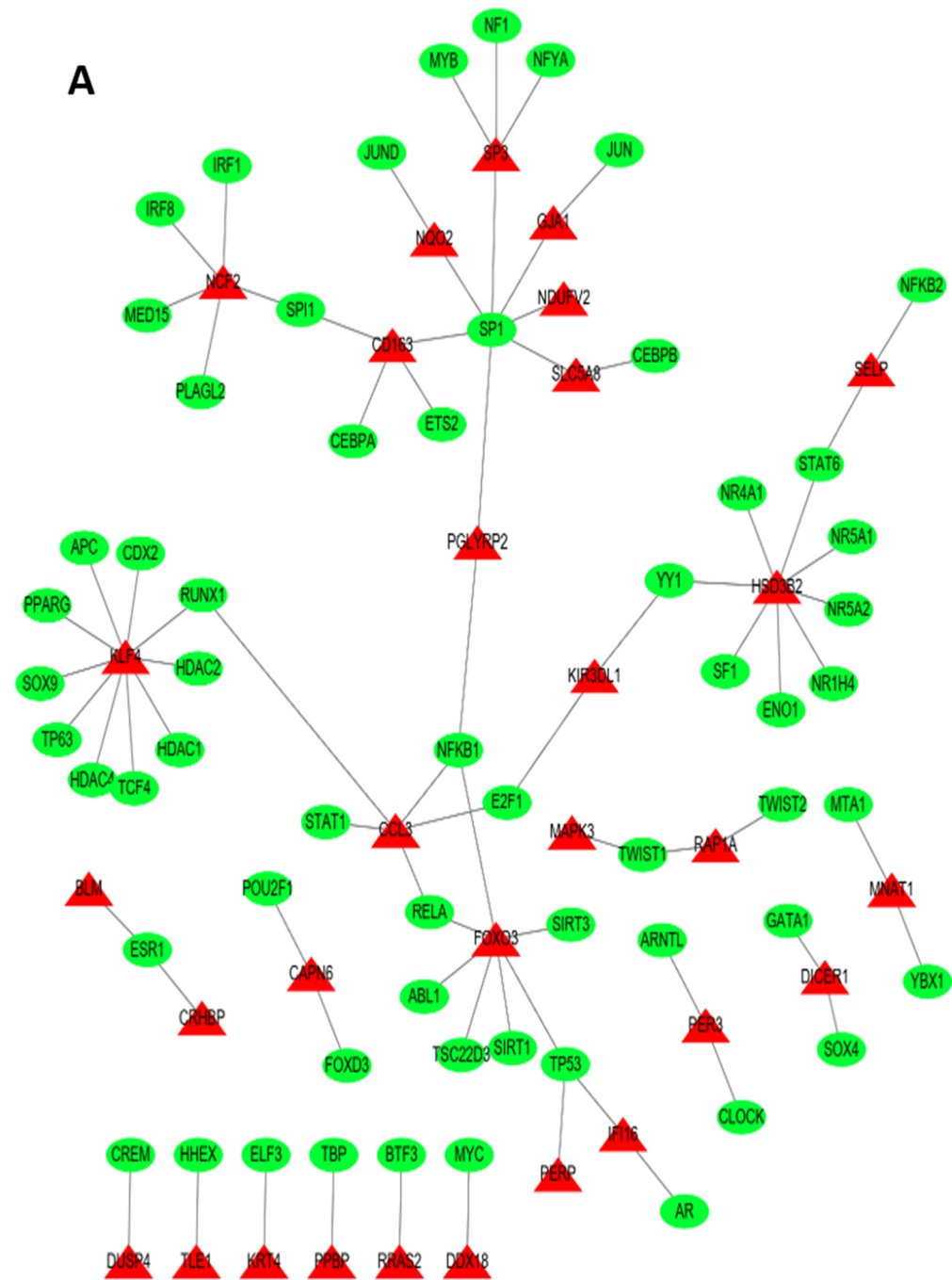

B

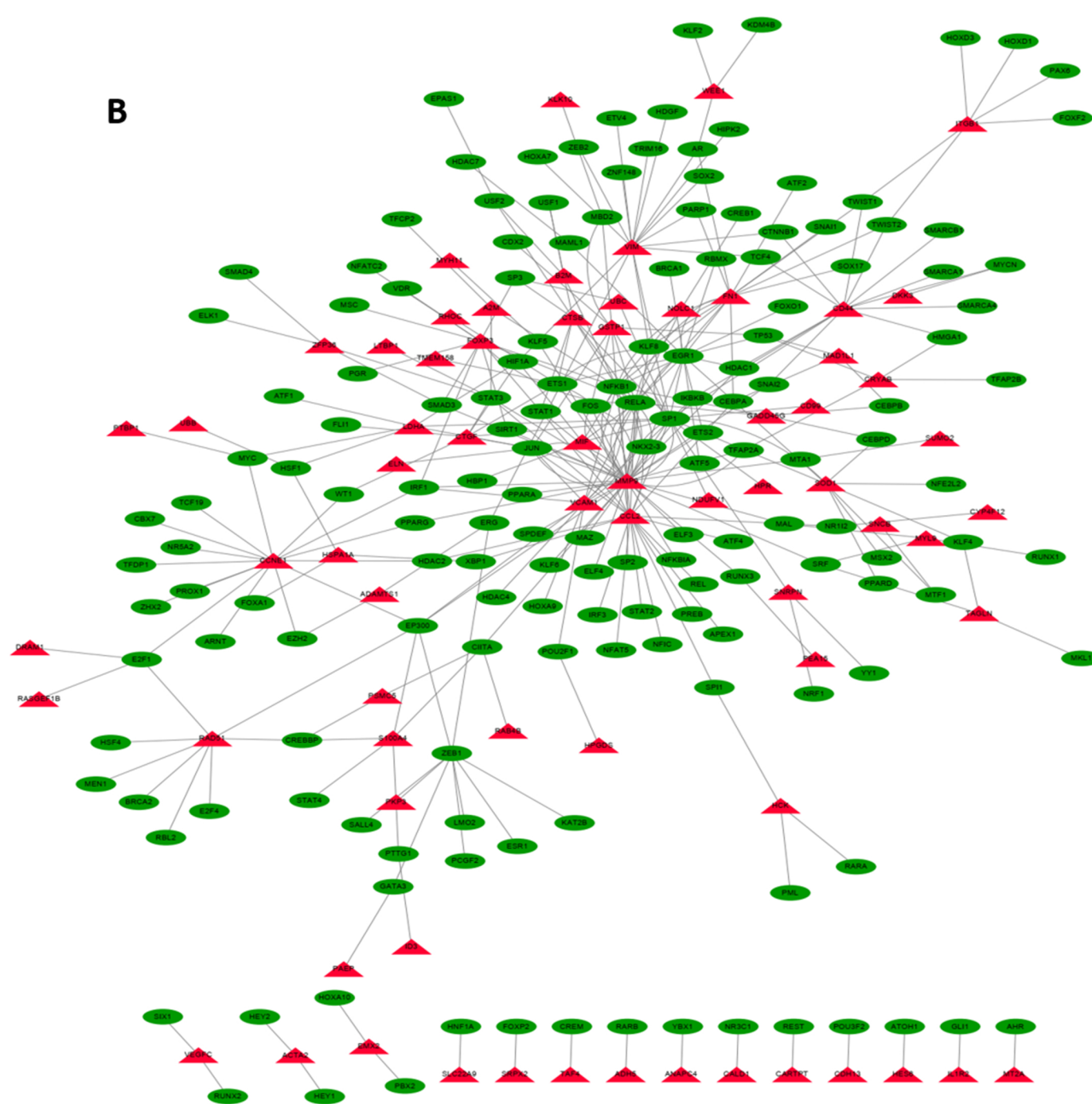

Supplement: Supplementary 6 — Construction of the transcriptional regulatory network of all genes in the hub module (A-B). (A) The transcriptional regulatory network of the tan module. (B) The transcriptional regulatory network of the yellow module. [file 5544276.f6.pdf]

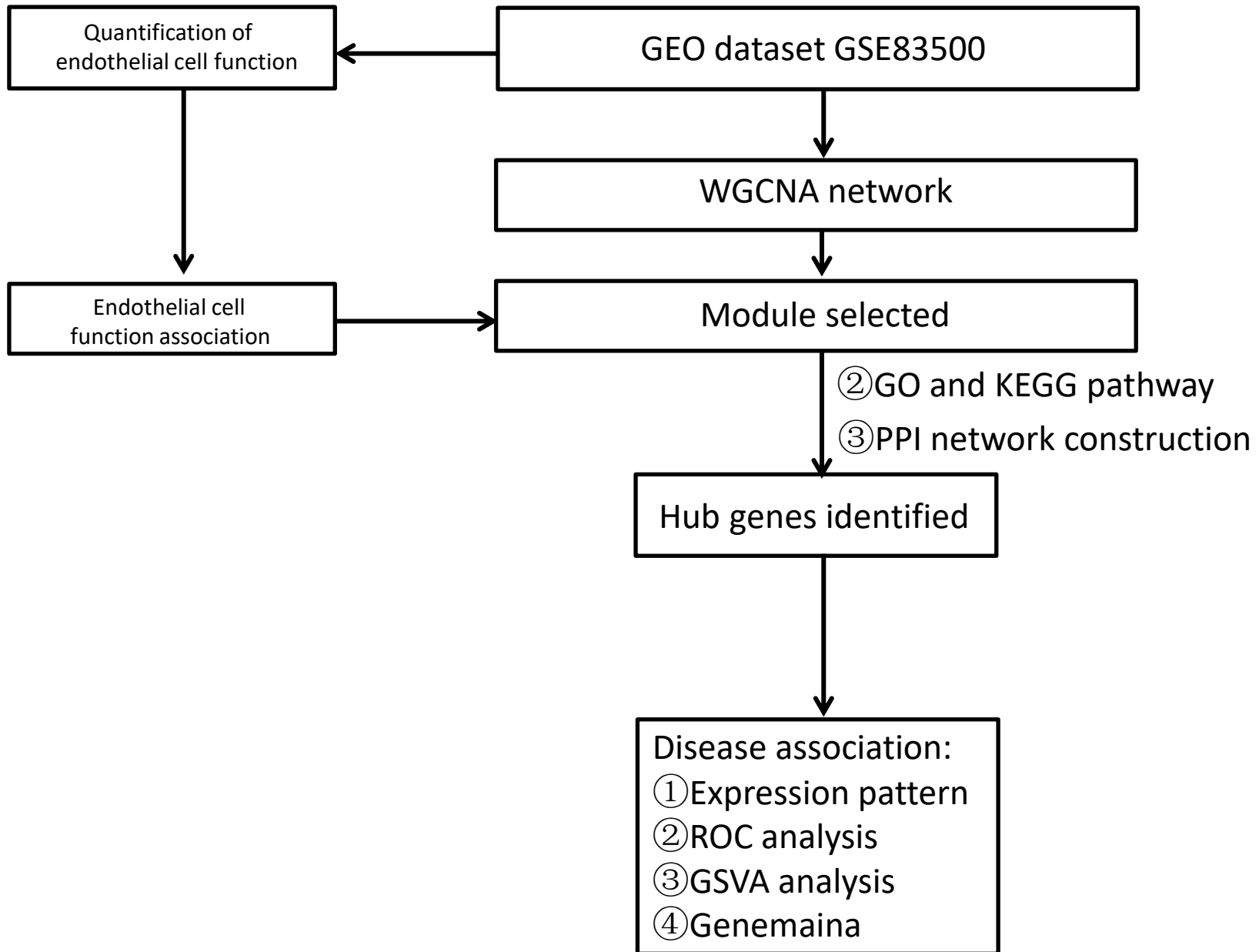

Supplement: Supplementary 7 — Flow chart of this experiment. [file 5544276.f7.pdf]
