# Supplementary material for: Identification of Key Genes Associated with Endothelial Cell Dysfunction in Atherosclerosis Using Multiple Bioinformatics Tools
Source: Biomed Res Int. 2022 Jan 10;2022:5544276. doi: 10.1155/2022/5544276 (PMC8764276; doi:10.1155/2022/5544276)
Supplement: Supplementary 8 — The protocol of Q-PCR in this study. [file 5544276.f8.pdf]

Protocol of Q-PCR

1: Extract sample total RNA

The Simple P Total RNA Extraction Kit (BioFlux, USA) was used to extract the total RNA of t he sample. The experimental operation was carried out according to the product manual.

The experimental operation steps are as follows:

1. Grind the tissue sample with a mortar, weigh 30mg of tissue powder and put it into a 1.5ml centrifuge tube;

2. Add 600μL to the pretreated sample cracking solution R2, fully reverse and mix well, and stand at room temperature for 3-5min;

3. Suck the supernatant into a purification column sleeved with a liquid receiving tube and centrifuge at 12000rpm for 30s;

4. Discard the liquid in the liquid receiving pipe and add 600μL washing solution to the purification column, centrifuged at 12000rpm for 30s;

5. Discard the liquid in the liquid receiving pipe and wash it again;

6. The empty column was centrifuged at 10000rpm for 1min, and then the purified column was transferred to a new 1.5ml centrifuge tube;

7. Add eluent 30-50μL in the center of purification column membrane. Total RNA was obtained by standing at room temperature for 1 min and centrifugation at 12000rpm for 30s;

8. Measure the concentration and store at -80℃.

2: Reverse transcription

With PrimeScript™ RT reagent Kit with gDNA Eraser (Perfect Real Time)for reverse transcription. The experimental operation was carried out according to the product manual.

The steps are as follows:

Add the first part of 10ul reaction system into the reaction tube according to the following table and mix well.

Reagent	10μl reaction system
5×gDNA Eraser Buffer	2 μl
gDNA Eraser	1 μl
Total RNA	3 μl
RNase Free H2O	4 μl

After heating at 42 ℃ for 2 minutes, place it at 4 ℃, centrifuge briefly, collect the reaction solution, and add the following components:

Reagent	20μl reaction system
Mixture	10μl
PrimeScript RT Enzyme Mix I	1μl
RT Primer Mix	1μl
5× PrimeScript Buffer 2 (for Real Time)	4μl
RNase Free dH2O	4μl

The cDNA was obtained by heating at 37 ℃ for 15min, inactivating at 85 ℃ for 5S and placing at 4 ℃.

3: Q-PCR

Instrument and analysis method

The model of fluorescence quantitative PCR instrument is Applied Biosystems® QuantStudio® 5. Use $2^{-\triangle\triangle CT}$ method for relative quantitative analysis of data.

The operation process is as follows:

Reaction system: the experimental operation shall be carried out according to the product manual.

Reagent	25μl reaction system
TB Green Premix Ex Taq II（Tli RNaseH Plus）(2×)	12.5μl
PCR Forward Primer(10μM)	1μl
PCR Reverse Primer(10μM)	1μl
RT reaction solution (cDNA solution)	2μl
RNase-Free ddH2O	8.5μl

Reaction procedure:

Cycles	Temperature	Time
1×	95℃	15min
40×	95℃	10s
	60℃	30s
