# Supplementary material for: Identification of Key Genes Associated with Endothelial Cell Dysfunction in Atherosclerosis Using Multiple Bioinformatics Tools
Source: Biomed Res Int. 2022 Jan 10;2022:5544276. doi: 10.1155/2022/5544276 (PMC8764276; doi:10.1155/2022/5544276)
Supplement: Supplementary 9 — The instructions of Lipofectamine® 2000 Reagent (Invitrogen, USA). [file 5544276.f9.pdf]

Lipofectamine® 2000 Reagent

invitrogen by life technologies™

Package Contents

Catalog Number	Size
11668-030	0.3 mL vial
11668-027	0.75 mL vial
11668-019	1.5 mL vial
11668-500	15 mL vial

Storage Conditions

Store at 4°C (do not freeze).

Required Materials

- Plasmid DNA (0.5–5 µg/µL stock)
- Opti-MEM® Reduced Serum Medium
- Eppendorf tubes

Timing

Preparation: 10 minutes
Incubation: 5 minutes
Final Incubation: 1–3 days

Selection Guide

Lipofectamine® Reagents

Go online to view related products.

Product Description

- Lipofectamine® 2000 Reagent is a proprietary formulation for transfecting nucleic acids into a wide range of eukaryotic cells.

Important Guidelines

- DNA-Lipofectamine® 2000 complexes must be made in serum-free medium such as Opti-MEM® Reduced Serum Medium and can be added directly to cells in culture medium, in the presence or absence of serum/antibiotic.
- It is not necessary to remove complexes or change/add medium after transfection.
- The amount of Lipofectamine® 2000 Reagent required for successful transfection varies depending on the cell type and passage number. Start any new transfection by testing the recommended four concentrations of Lipofectamine® 2000 Reagent to determine an optimum amount.

Online Resources

Visit our product page for additional information and protocols. For support, visit www.lifetechnologies.com/support.

For Research Use Only. Not for use in diagnostic procedures.

Print Options

Protocol Outline

A. Plate cells so they will be 70–90% confluent at the time of transfection.
B. Prepare plasmid DNA-lipid complexes.
C. Add DNA-lipid complexes to cells.

Lipofectamine® 2000 DNA Transfection Reagent Protocol

See page 2 to view a typical DNA transfection procedure.

Component	96-well	24-well	6-well
Final DNA per well	100 ng	500 ng	2500 ng
Final Lipofectamine® 2000 Reagent per well	0.2–0.5 µL	1.0–2.5 µL	5.0–12.5 µL

Co-Transfection of Plasmid DNA and siRNA

Transfect plasmid DNA and siRNA at the same time using Lipofectamine® 2000 Reagent by adding 30 pmol (~0.6 µg) of siRNA per 1 µg of DNA.

mRNA Transfection

mRNA can be transfected in a 24-well plate using Lipofectamine® 2000 Reagent by adding 0.5–1 µg of mRNA per well.

Photograph of Expected Results

Scaling Up or Down Transfections

Limited Product Warranty and Disclaimer Details

life technologies™

Lipofectamine® 2000 DNA Transfection Reagent Protocol

Transfect cells according to the following chart. Volumes are given on a per-well basis. **Each reaction mix is sufficient for triplicate (96-well), duplicate (24-well), and single well (6-well) transfections, and accounts for pipetting variations.** Adjust the amounts of components according to your tissue culture format. For additional information on scaling your transfection reaction, see page 1.

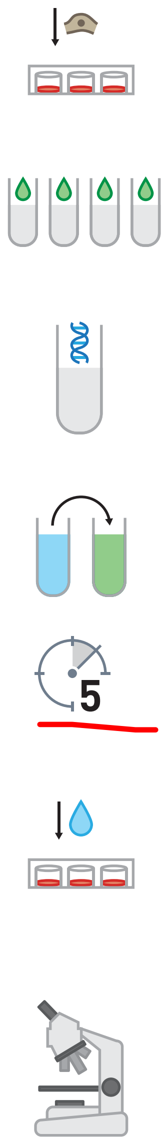

Timeline		Steps	Procedure Details: Component	96-well	24-well	6-well
Day 0	1	Seed cells to be 70–90% confluent at transfection	Adherent cells	1–4 × 10^4	0.5–2 × 10^5	0.25–1 × 10^6
Day 1	2	Dilute four amounts of Lipofectamine® Reagent in Opti-MEM® Medium	Opti-MEM® Medium	25 µL × 4	50 µL × 4	150 µL × 4
			Lipofectamine® 2000 Reagent	i 1, 1.5, 2, 2.5 µL	i 2, 3, 4, 5 µL	i 6, 9, 12, 15 µL
	3	Dilute DNA in Opti-MEM® Medium	Opti-MEM® Medium	125 µL	250 µL	700 µL
			DNA (0.5–5 µg/µL)	2.5 µg	5 µg	14 µg
	4	Add diluted DNA to diluted Lipofectamine® 2000 Reagent (1:1 ratio)	Diluted DNA Total	25 µL	50 µL	150 µL
			Diluted Lipofectamine® 2000 Reagent	25 µL	50 µL	150 µL
	5	Incubate	Incubate for 5 minutes at room temperature.			
			**Component**	**96-well**	**24-well**	**6-well**
	6	Add DNA-lipid complex to cells	DNA-lipid complex per well	10 µL	50 µL	250 µL
			Final DNA used per well	100 ng	500 ng	2500 ng
			Final Lipofectamine® 2000 Reagent used per well	0.2–0.5 µL	1.0–2.5 µL	5.0–12.5 µL
Day 2–4	7	Visualize/analyze transfected cells	Incubate cells for 1–3 days at 37°C. Then analyze transfected cells.			

12 June 2013

For support, visit www.lifetechnologies.com/support.
