# Supplementary material for: Identification of Key Genes Associated with Endothelial Cell Dysfunction in Atherosclerosis Using Multiple Bioinformatics Tools
Source: Biomed Res Int. 2022 Jan 10;2022:5544276. doi: 10.1155/2022/5544276 (PMC8764276; doi:10.1155/2022/5544276)
Supplement: Supplementary 10 — Short description of GSE83500\GSE28829\GSE43292 datasets. [file 5544276.f10.zip › 10-1Short description of GSE83500 dataset (1).pdf]

Short description of GSE83500 dataset

	Title	Sex	Age	Race	Extracted molecule	Organism
**1**	MI patient 1	Male	69	Malay	total RNA	Homo sapiens
**2**	MI patient 2	Male	56	Chinese	total RNA	Homo sapiens
**3**	MI patient 3	Male	58	Chinese	total RNA	Homo sapiens
**4**	MI patient 4	Female	81	Chinese	total RNA	Homo sapiens
**5**	MI patient 5	Female	68	Chinese	total RNA	Homo sapiens
**6**	MI patient 6	Male	64	Chinese	total RNA	Homo sapiens
**7**	MI patient 7	Male	50	Chinese	total RNA	Homo sapiens
**8**	MI patient 8	Male	49	Malay	total RNA	Homo sapiens
**9**	MI patient 9	Male	55	Chinese	total RNA	Homo sapiens
**10**	MI patient 10	Male	64	Malay	total RNA	Homo sapiens
**11**	MI patient 11	Male	52	Chinese	total RNA	Homo sapiens
**12**	MI patient 12	Male	56	Chinese	total RNA	Homo sapiens
**13**	MI patient 13	Male	53	Chinese	total RNA	Homo sapiens
**14**	MI patient 14	Male	54	Indian	total RNA	Homo sapiens
**15**	MI patient 15	Male	63	Chinese	total RNA	Homo sapiens
**16**	MI patient 16	Male	70	Indian	total RNA	Homo sapiens
**17**	MI patient 17	Male	63	Chinese	total RNA	Homo sapiens
**18**	non-MI patient 1	Male	56	Chinese	total RNA	Homo sapiens
**19**	non-MI patient 2	Male	53	Chinese	total RNA	Homo sapiens
**20**	non-MI patient 3	Male	58	Chinese	total RNA	Homo sapiens
**21**	non-MI patient 4	Male	70	Chinese	total RNA	Homo sapiens
**22**	non-MI patient 5	Male	50	Chinese	total RNA	Homo sapiens
**23**	non-MI patient 6	Female	61	Chinese	total RNA	Homo sapiens
**24**	non-MI patient 7	Male	63	Chinese	total RNA	Homo sapiens
**25**	non-MI patient 8	Male	56	Other	total RNA	Homo sapiens
**26**	non-MI patient 9	Male	65	Chinese	total RNA	Homo sapiens
**27**	non-MI patient 10	Male	62	Chinese	total RNA	Homo sapiens
**28**	non-MI patient 11	Female	81	Chinese	total RNA	Homo sapiens
**29**	non-MI patient 12	Male	78	Indian	total RNA	Homo sapiens
**30**	non-MI patient 13	Male	56	Malay	total RNA	Homo sapiens
**31**	non-MI patient 14	Female	52	Malay	total RNA	Homo sapiens
**32**	non-MI patient 15	Male	55	Indian	total RNA	Homo sapiens
**33**	non-MI patient 16	Male	48	Malay	total RNA	Homo sapiens
**34**	non-MI patient 17	Male	57	Malay	total RNA	Homo sapiens
**35**	non-MI patient 18	Male	52	Malay	total RNA	Homo sapiens
**36**	non-MI patient 19	Male	62	Chinese	total RNA	Homo sapiens
**37**	non-MI patient 20	Male	61	Malay	total RNA	Homo sapiens

The method of dataset formation

1. Ascending aortic wall punch biopsies obtained as a standard part of coronary artery bypass surgery. A total of 37 (17 MI, 20 Non-MI) frozen aortic tissues were embedded in TissueTek optimal cutting temperature compound.

2. The embedded aortic tissues were trimmed and sectioned to a thickness of 10µm and placed on an RNase-free Polyethylene Naphthalate membrane slide . Each slide containing frozen aortic sections was stained with Arcturus Histogene LCM Frozen Section Staining Kit according to the manufacturer's protocol to enhance the visibility of VSMCs – elongated and spindle-shaped.

3. The dissected VSMCs were scraped into a microcentrifuge tube containing 100µL of ice-cold TRI Reagent® , with 19G sterile needles and freeze down in dry ice.

4. Total RNA was isolated from VSMCs with Tri Reagent® following manufacturer's protocol and amplified with Ovation FFPE WTA System .

5. The cDNA products were further subjected to fragmentation and labelling with Encore Biotin Module . Hybridize to the Human Genome U219 Plus 2.0 Array plate. All the reaction plates were washed, stained and scanned with the Gene Titan Instrument. Gene expression data from laser captured microdissection of vascular smooth muscle cells .

6. Samples analyzed with Affymetrix platform were preprocessed using the Raw CEL data sets of all samples were normalized by Robust Multi-array Average algorithm using the R package affy.
