# Supplementary material for: Identification of Key Genes Associated with Endothelial Cell Dysfunction in Atherosclerosis Using Multiple Bioinformatics Tools
Source: Biomed Res Int. 2022 Jan 10;2022:5544276. doi: 10.1155/2022/5544276 (PMC8764276; doi:10.1155/2022/5544276)
Supplement: Supplementary 10 — Short description of GSE83500\GSE28829\GSE43292 datasets. [file 5544276.f10.zip › 10-2Short description of GSE28829 dataset (1).pdf]

Status	Public on Apr 25, 2011
Title	advanced atherosclerotic plaque1
Sample type	RNA
Source name	postmortem plaque
Organism	Homo sapiens
Characteristics	tissue: carotid artery<br>phenotype: advanced atherosclerotic plaque
Extracted molecule	total RNA
Extraction protocol	Guanidine isothiocyanate/CsCl method followed by further purification and concentration using RNease mini columns (Qiagenm Hilden, Germany)
Label	Biotin
Label protocol	GeneChip IVT Labeling Kit (Affymetrix, Santa Clara, USA, CA)
Hybridization protocol	Affymetrix Eukaryotic Target Hybridization protocol (GeneChip Expression Analysis Technical Manual)
Scan protocol	GeneChip Scanner 3000 (Affymetrix, Santa Clara, USA, CA)
Description	Advanced atherosclerotic plaque from human carotid (autopsy)
Data processing	Probe set summarization and normalization was performed by robust multi-array averaging (RMA, including background subtraction, summarization by median polish and quantile normalization) using the R/Bioconductor package affy.

Submission date	Apr 25, 2011
Last update date	Apr 25, 2011
Contact name	Marco Manca
E-mail(s)	marco.manca@cern.ch
Organization name	University of Maastricht
Department	CARIM
Lab	Experimental Vascular Pathology
Street address	P. Debyelaan 25
City	Maastricht
State/province	Limburg
ZIP/Postal code	6229 HX
Country	Netherlands

Platform ID	GPL570
