# Supplementary material for: Identification of Key Genes Associated with Endothelial Cell Dysfunction in Atherosclerosis Using Multiple Bioinformatics Tools
Source: Biomed Res Int. 2022 Jan 10;2022:5544276. doi: 10.1155/2022/5544276 (PMC8764276; doi:10.1155/2022/5544276)
Supplement: Supplementary 10 — Short description of GSE83500\GSE28829\GSE43292 datasets. [file 5544276.f10.zip › 10-3Short description of GSE43292dataset (1).pdf]

Status	Public on Apr 01, 2013
Title	Genome-wide expression study of human carotid atheroma
Organism	Homo sapiens
Experiment type	Expression profiling by array
Summary	The aim of this study was to identify new biomarkers and to investigate pathways involved in the progression of human carotid atheroma.
Overall design	The study was conducted from pieces of carotid endarterectomy collected in 32 hypertensive patients. The samples contained media and neo-intima without adventitia. They were paired, including for each patient one sample of the atheroma plaque (stage IV and over of the Stary classfication) containing core and shoulders of the plaque, and one sample of distant macroscopically intact tissue (stages I and II). In addition, clinical, biological and histological data were collected.
Contributor(s)	Bricca G, Ayari H, Legedz L, Cerutti C
Citation(s)	• Ayari H, Bricca G. Identification of two genes potentially associated in iron-heme homeostasis in human carotid plaque using microarray analysis. *J Biosci* 2013 Jun;38(2):311-5. PMID: 23660665
Submission date	Jan 04, 2013
Last update date	Jul 26, 2018
Contact name	Catherine Cerutti
E-mail(s)	cerutti@univ-lyon1.fr
Organization name	Université Lyon 1 & Hôpital Nord-Ouest
Lab	EA4173 Génomique Fonctionnelle de l'Hypertension Artérielle
Street address	8 avenue Rockefeller
City	Lyon
ZIP/Postal code	69373
Country	France
Platforms (1)	GPL6244 [HuGene-1_0-st] Affymetrix Human Gene 1.0 ST Array [transcript (gene) version]
